# Dietary Patterns and Fertility

**DOI:** 10.3390/biology13020131

**Published:** 2024-02-19

**Authors:** Martina Cristodoro, Enrica Zambella, Ilaria Fietta, Annalisa Inversetti, Nicoletta Di Simone

**Affiliations:** 1 Department of Biomedical Sciences, Humanitas University, Via Rita Levi Montalcini 4, Pieve Emanuele, 20072 Milano, Italy; 2 IRCCS Humanitas Research Hospital, Via Manzoni 56, 20089 Rozzano, Italy

**Keywords:** nutrition, Mediterranean diet, Western diet, macronutrients, fertility status

## Abstract

**Simple Summary:**

The link between diet and fertility has already been unraveled. Lifestyle, in particular a healthy diet, may improve fertility both in men and in women. Worldwide diet has changed, and this condition may partially explain the reduced global fertility rate. This review analyzes the principal different dietary patterns and their influences on fertility. Specifically, the Mediterranean diet seems to have a positive influence on fertility, while the Western diet and Westernized diets (such as Middle Eastern and Asian diets) seem to have a negative influence on fertility. Summarizing the results, a diet rich in saturated fatty acids, cholesterol, animal proteins, and carbohydrates with a high glycemic index is strictly correlated with male and female infertility. On the contrary, a diet rich in plant proteins, vegetables, fruits, and antioxidants (carotenoids, vitamin C, vitamin E, flavonoids, and polyphenols) may improve fertility. The examination of the molecular mechanisms by which different diets impact fertility may lead to more personalized treatments in infertile couples. Moreover, these results may encourage public health policies that promote healthy dietary patterns.

**Abstract:**

Diet has a key role in the reproductive axis both in males and females. This review aims to analyze the impacts of different dietary patterns on fertility. It appears that the Mediterranean diet has a predominantly protective role against infertility, while the Western diet seems to be a risk factor for infertility. Moreover, we focus attention also on dietary patterns in different countries of the World (Middle Eastern diet, Asian diet). In particular, when analyzing single nutrients, a diet rich in saturated fatty acids, cholesterol, animal proteins, and carbohydrates with high glycemic index is highly associated with male and female infertility. Finally, we evaluate the effects of vegetarian, vegan, and ketogenic diets on fertility, which seem to be still unclear. We believe that comprehension of the molecular mechanisms involved in infertility will lead to more effective and targeted treatments for infertile couples.

## 1. Introduction

Infertility is defined as the failure to achieve a clinical pregnancy after 12 months or more of regular unprotected sexual intercourse in women younger than 35 years old. According to the American College of Obstetricians and Gynecologists (ACOG), in women older than 35 years old an evaluation is warranted after 6 months of unprotected intercourse. This condition affects 15% of couples in Italy and one in six people worldwide [1].

Lifestyle seems to have a major impact on reproductive health, in terms of body weight, body composition, physical activity, and nutrient intake [2,3]. Specifically, both quantitative and qualitative dietary characteristics affect fertility. It has been shown that unhealthy diets, which are either high in calories or low in calories, can disrupt the physiological processes involved in reproduction, such as ovulatory and sperm capacitation, and significantly increase the risk of infertility [4]. In particular, on one side, Body Mass Index (BMI) lower than 18 kg/m^2^ (in an underweight population) is associated with chronic energy deficiency, which affects the gonadotropin-releasing hormone (GnRH) pulse generator. On the other side, obesity affects reproductive function at a metabolic level: insulin resistance and hyperinsulinemia seem to be correlated with high levels of luteinizing hormone (LH), reversal of the LH/FSH ratio [5,6], and enhanced steroidogenesis [7,8].

Another factor that explains the impact of diet on fertility is the fact that nutrients exert a bioactive role, influencing fertility both in males and in females [9].

Considering fatty acids, Trans Fatty Acids (TFA) promote insulin resistance and increase inflammatory markers; these mechanisms can lead to infertility [10,11,12]. The role of Polyunsaturated Fatty Acids (PUFAs) on fertility is still debated. Some studies showed that PUFAs have beneficial effects on fertility by reducing the risk of anovulation and increasing progesterone concentrations [13,14]. Others concluded that PUFAs are correlated with a reduced fertility rate [15,16]. Lastly, Monounsaturated Fatty Acids (MUFAs) have been positively associated with fertility; they can bind to peroxisome proliferator–activated receptor γ (PPAR-γ), reducing inflammation [13,16]. 

The quantity and quality of carbohydrates in the diet may have impacts on reproductive processes. A correlation has been found between reduced insulin sensitivity and both reduced levels of Sex Hormone Binding Globulin (SHBG) and reduced androgen synthesis in PCOS and diabetic patients. On the contrary, the consumption of products with a high glycemic index may increase insulin resistance, dyslipidemia, and oxidative stress, which are factors that reduce fertility [17,18].

Depending on the protein source, dietary protein intake appears to have opposite effects on fertility; while animal proteins seem to be associated with an increased risk of infertility, plant proteins seem to improve fertility [19]. 

**Figure 1:**
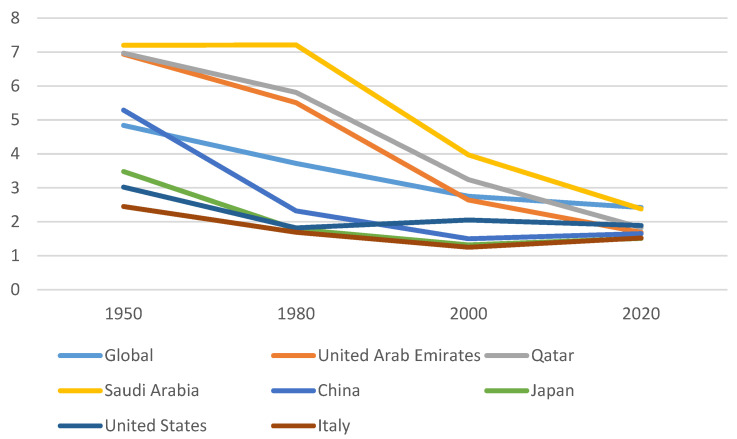
TFR in some countries in the World [22].

The total fertility rate (TFR) is the average number of children that women would have if they were to live until the end of the reproductive period and if they were subject to the current age-specific fertility rates. Nowadays, globally the total fertility rate (TFR) is 2.3 births per woman. In 1950 TFR was around five. It is estimated that in 2050 the TFR will be around 2.1 [20]. This decline is linked to different factors; among them, high-calorie food and more sedentary lifestyles have a great impact on fertility both in high-income countries and in low-income countries [21]. It is interesting to analyze how the TFRs of principal countries have changed in the last 70 years (Figure 1). Globally, the TFR was 4.84 in 1950; then it became 3.72 in 1980, 2.75 in 2000, and 2.42 in 2020 [22]. Among the Arab States, in the United Arab Emirates the TFR varied from 6.94 (in 1950) to 5.51 (in 1980), to 2.64 (in 2000), and to 1.69 (in 2020). Also, in Qatar the TFR changed drastically: it was 6.97 in 1950, 5.81 in 1980, 3.24 in 2000, and 1.83 in 2020. Moreover, in Saudi Arabia, the TFR was 7.20 in 1950, 7.21 in 1980, 3.97 in 2000, and 2.38 in 2020. Among Asian States, in China the TFR declined: firstly, it was 5.29 (in 1950), then it was 2.32 (in 1980), 1.50 (in 2000), and 1.65 (in 2020). Analyzing Japan, the TFR was 3.48 in 1950, 1.78 in 1980, 1.32 in 2000, and 1.51 in 2020. Moving our attention to the Western world, in the United States the TFR decreased from 3.02 in 1950, to 1.82 in 1980, 2.05 in 2000, and 1.89 in 2020. In Italy, the TFR changed from 2.45 in 1950 to 1.69 in 1980, 1.25 in 2000, and 1.52 in 2020 [22].

How does diet influence fertility and TFR?

## 2. Mediterranean Diet

The Mediterranean diet (MedDiet) is a diet based on the traditional foods of the countries that surround the Mediterranean Sea, including Italy, Greece, and Spain [23]. This term was coined for the first time in 1960 by Ancel Keys who found, in an epidemiological study, a lower incidence of cardiovascular diseases and cancer in the populations of countries along the Mediterranean Sea [24]. This diet is characterized by daily consumption of fruits and vegetables, legumes, whole grains, nuts, and olive oil (especially virgin and extra virgin olive oil [EVOO]), which represent the major sources of fats [25]. At the same time, the diet involves moderate consumption of dairy products, fish, poultry, and wine, and lastly, limited consumption of red- and processed meats and sweets [25]. 

In recent years, evidence has been acquired about the beneficial roles of the MedDiet in cardiovascular diseases and diabetes. Its effects on human fertility represent a field of increasing interest. Regarding female fertility, different studies demonstrated a higher percentage of clinical pregnancy and live births and more viable embryos in patients undergoing assisted reproductive technologies (ART) with higher MedDiet adherence [26,27]. Moreover, a prospective study demonstrated that a dietary pattern like the MedDiet was positively related to folate and vitamin B6 levels in the blood and follicular fluid, with an increase of 40% in pregnancy rate after intracytoplasmic sperm injection (ICSI) [28]. On the other hand, a recent meta-analysis by Yang et al. found no significant positive association between the MedDiet and successful implantation, clinical pregnancy, or live birth following IVF [29]. Similar results were obtained in a recent prospective study in which there was no significant association between adherence to the MedDiet and successful clinical pregnancy in women undergoing ART [30]. For patients trying to conceive spontaneously, higher adherence to the MedDiet seems to be associated with a reduced interval to conception [31]. 

Regarding male fertility, a meta-analysis by Su et al. demonstrated that men with greater compliance to healthy diets, including the MedDiet, had better sperm parameters in terms of sperm concentration, progressive sperm motility, and total sperm count compared to those with poorer compliance [32]. Furthermore, Montano et al. demonstrated higher sperm concentration, total motility, and progressive after 4 months of the MedDiet, and a reduced proportion of spermatozoa with abnormal morphology [33]. Finally, a recent observational cross-sectional study found that males with the highest Mediterranean Diet adherence showed a higher probability of normozoospermia, while patients in the lower-MedDiet-adherence group showed at least one sperm alteration in more than 90% of cases [34]. 

In the following paragraphs, the macronutrients and micronutrients specific to the MedDiet and their relationships with female and male fertility are illustrated, with a focus on fruit and vegetables.

### 2.1. Typical Foods of Mediterranean Diet

#### 2.1.1. Fats

There are several examples of evidence of the beneficial effects of typical fat components found in the MedDiet, which are probably due to changes in anti-inflammatory responses. 

The MedDiet is linked to higher tissue levels of polyunsaturated fatty acids (PUFAs), that act as precursors of the anti-inflammatory eicosanoids, which are involved in platelet aggregation and the regulation of inflammatory responses [35,36]. Moreover, the uterine microenvironment in patients with repeated implantation failure is lacking in PUFAs, which may have an impact on endometrial functions [37]. Ω-3 PUFA (eicosapentaenoic and docosahexaenoic acid), found mainly in fatty fish, was related to a positive outcome in women undergoing ART treatment, showing a significant association with embryo morphology [38]. In males, ω-3 fatty acids act to reduce the risk of asthenozoospermia, improving normal sperm morphology, increasing total sperm count, concentration, motility, and volume, and reducing sperm DNA fragmentation [39]. Their role in the regulation of membrane fluidity, spermatogenesis, and sperm motility could be considered predictors of cryopreservation success [40].

Even EVOO has a fundamental role in fertility. One randomized clinical trial reported better embryo development in patients who received supplementation with EVOO than in patients who received sunflower oil or placebo drinks [41].

#### 2.1.2. Carbohydrates

As mentioned above, carbohydrates such as whole grains represent an important brick of the Mediterranean food pyramid. The impact of these nutrients on fertility is still debated. 

Considering whole grains, the correlation between whole grain intake and live birth rate is controversial. Gaskin et al. demonstrated a positive correlation between whole grain intake and live birth rate, with a likelihood of 53% (95% CI: 41, 65) in the highest quartile (>52.4 g/day) compared to 35% (95% CI: 24, 46) in the lowest quartile of intake (>21.4 g/day) [42]. On the other hand, another study reported no association between whole grain consumption and live birth in patients undergoing ART [43]. 

Corsetti et al. reported that consumption of a low-carb MedDiet for three months is linked to lower sperm DNA fragmentation and increased testosterone levels [44]. The rationale is that reduced glycemic index, reduced hyperinsulinemia, and reduced insulin resistance have a positive effect on male fertility [45]. 

#### 2.1.3. Proteins

The MedDiet includes the regular consumption of vegetal protein and a reduced intake of animal protein, preferring dairy products, fish, and poultry. In one study, women who replaced 5% of their animal protein intake with vegetable protein had a 50% reduced risk of ovulatory infertility, while another study reported no association between vegetable sources of protein (beans, nuts, and soy) and implantation, clinical pregnancy, and live birth rate after ART [19,46]. In a cohort study, a higher intake of food enriched in fish and white meat was positively associated with the chance of blastocyst formation versus a negative correlation with red meat [47]. 

The negative effect of red meat on embryo development and pregnancy could be explained by the absorption of advanced glycation end products (AGE), derived from the cooking process of animal-derived food. AGE can cause intracellular damage leading to infertility both in males and females. AGE accumulation causes oxidative stress, both in oocyte and sperm cells. In women, oxidative stress damages oocyte DNA, accelerating ovarian aging. This condition can determine increased follicle apoptosis and decreased ovarian function. In men, oxidative stress damages sperm DNA, altering both sperm motility and sperm capacitation. Moreover, increased levels of ROS damage the testis, blocking spermatogenesis [48]. 

#### 2.1.4. Micronutrients

Vitamin E is commonly found in plant-based oils, nuts, seeds, fruits, and vegetables. Vitamin C (ascorbic acid) is found in many fresh fruits like oranges, lemons, limes, grapefruit, cantaloupes, mangoes, papayas, and their juices. These two vitamins are known to be potent free radical scavengers and antioxidants [49] and various studies have demonstrated improved seminal quality with ascorbic acid supplementation [50]. 

A lipophilic antioxidant carotenoid frequently found in tomatoes and several red fruits, which are commonly present in the MedDiet, is lycopene. This molecule is a modulator of lipid peroxidation, antioxidant enzyme activities, and Krebs cycle enzyme functions; for this reason, it has a positive effect on testicular mitochondrial function and sperm quality [51].

Other micronutrients commonly found in plant-based foods, berries, fish, and grain carbohydrates are flavonoids and polyphenols, which are considered the key constituents of anti-inflammatory diets [52]. Flavonoids could be involved in the downregulation of inflammatory pathways in different way: by scavenging free radicals such as reactive oxygen species (ROS), inhibiting the key inflammatory signaling pathways, and up regulating detoxifying enzymes [53]. Rutin, quercetin, and epigallocatechin are flavonoids that have been shown to improve sperm motility, plasma, and acrosomal membrane integrity, and to lower intracellular ROS concentration of frozen sperm [54]. Similarly, carotenoids and polyphenols act as potent scavengers of ROS, inhibit lipid peroxidation, and influence the transcription of factors involved in the upregulation of pro-inflammatory cytokines [55]. 

Lastly, resveratrol found in grapes is a well-known cytoprotective substance that has been demonstrated to increase total and progressive sperm motility, restore chromatin compactness, and decrease sperm lipoperoxidation in vitro [56].

#### 2.1.5. Fruit and Vegetables

According to the MedDiet, daily portions of fruit and vegetables are needed. 

In a prospective cohort study by Grieger et al., it was demonstrated that women consuming fruit less than three times per month had an increased risk of 29% for infertility (RR (95% CI): 1.29 (0.95, 1.74)) compared to 7% (RR (95% CI): 1.07 (0.88, 1.29)) in patients consuming fruit more than three times per day [57]. Similarly, Qu et al. reported a two-fold higher risk of stillbirth in Chinese patients with a low appetite for vegetables who had a spontaneous pregnancy (OR (95% CI): 1.99 (1.00, 3.93)) [58]. Interestingly, according to the results from a recent meta-analysis on the associations between dietary patterns and miscarriage, a high intake of fruit and vegetables is related to a reduction in miscarriage odds of 61% and 41%, respectively (OR (95% CI): 0.39 (0.33, 0.46); OR (95% CI): 0.59 (0.46, 0.769)) [59]. The other two studies reported similar results, finding a significant difference in fruit and vegetable consumption between patients who had live births and patients who experienced miscarriage or premature birth with fetal weight <2.5 kg [60,61]. On the other hand, other studies reported no differences in the combined intake of fruit and vegetables between fertile and infertile women and no effect of fruit and vegetables on live birth, clinical pregnancy, or implantation rate after ART [62,63]. In detail, Revonta et al. analyzed a population of 7021 people, including 155 infertile women and 289 infertile men. They demonstrated that infertile women did not significantly differ from fertile women in fruit and vegetable consumption in women aged 20–34 years (*p* = 0.34), in women aged 35–49 years (*p* = 0.13) and, finally, in women over 50 years (*p* = 0.35). Similarly, in men there was no statistically significant difference between fertile and infertile men, both in men aged 20–34 years (*p* = 0.93), in men aged 35–49 years (*p* = 0.57), and in men over 50 years (*p* = 0.15) [62]. 

## 3. Western Diet

The Western diet is a diet based on pre-packaged foods, red meat, industrially produced animal products, high-sugar drinks, candy and sweets, fried foods, butter, and other high-fat dairy products, eggs, and potatoes. Moreover, this diet is low in fruits and vegetables, whole grains, fish, nuts, and seeds [64]. This is the typical diet of developed countries, particularly the United States [65]. 

In contrast to the Mediterranean diet, the Western diet is unbalanced and implies an excess of calories [66]. In consequence, this dietary habit is frequently linked to obesity, which has a great impact on fertility both in females and in males [67,68]. Adipocytes secrete two key proteins: adiponectin and leptin [69,70,71]. Recently, Wu et al. demonstrated that in chickens adiponectin inhibits GnRH secretion via AMPK and PI3K signaling pathways [72]. The inhibition of GnRH secretion and pulsatility alters the reproductive axis leading to infertility [73]. Leptin has an impact on fertility, altering the steroidogenic pathway and reducing estrogen and progesterone production [74]. Moreover, leptin has a key role in the inflammatory state in the testicle [75], which is associated with an increased level of ROS [76]. High ROS levels are responsible for damage to cellular and mitochondrial membranes, leading to reductions in both in sperm motility and sperm concentration [77].

### 3.1. Typical Foods of the Western Diet

The impact of the Western diet on fertility depends on the amounts and the qualities of the foods introduced. We will discuss the single nutrients.

#### 3.1.1. Fats

In the Western diet, lipid overload can involve both cholesterol and fatty acids. In detail, the percentages of fats in the Western diet are 62.4% for saturated fatty acids (SFA), 30.7% for MUFA, and 6.9% for PUFA. Cholesterol represents only 1% of fats [78].

Cholesterol is generally found in eggs, shellfish, meat, and dairy products. Cholesterol is essential for membrane structure. Hypercholesterolemia alters membrane fluidity, which is fundamental for sperm motility and capacitation [79]. Moreover, higher levels of cholesterol cause the activation of endoplasmic reticulum stress in testicular Leydig cells. There is a downregulation of steroidogenic enzymes and, consequently, decreased testosterone production [80]. As reported before, reduced levels of testosterone lead to lower concentrations of spermatozoa. 

Fatty acids can be divided into TFAs, PUFAs, MUFAs, and SFAs. The Western diet, compared to the Mediterranean diet, is rich in SFAs. SFAs are generally found in butter, cheese, dairy desserts, meat products such as sausage and bacon, grain-based desserts (cookies), and fast food dishes. Excessive SFAs alter fertility in different ways. Firstly, fatty acids are essential for membrane structure. Exactly like hypercholesterolemia, increased levels of SFAs alter sperm membrane structure and consequently sperm motility and capacitation [79]. Moreover, recent studies demonstrated that SFAs also have a key role in the energy metabolism of spermatozoa. In detail, SFAs interact with sperm lactate dehydrogenase isoenzymatic form (LDH-C4), an enzyme that converts pyruvate to lactate resulting in the oxidation of NADH to NAD+ [81,82]. SFAs decrease LDH-C4 activity leading to reduced energetic metabolism and increased oxidative stress [83]. Moreover, SFAs also have a negative effect in women; higher levels of SFAs can cause ovulatory disorders [84]. Specifically, increased levels of SFAs are associated with increased insulin resistance, increased inflammatory marker concentrations, and a reduction in PPAR-γ expression; these are the mechanisms that negatively influence ovulation [85].

#### 3.1.2. Carbohydrates

Carbohydrates are present both in healthy foods, such as fruits and vegetables, and in unhealthy foods, such as sweets, French fries, and sugar-sweetened beverages. The Western diet is characterized by the consumption of simple carbohydrates, which have a high glycemic index (sugar and sugary foods, white bread, potatoes, and white rice) [48]. A high intake of carbohydrates with a high glycemic index is associated with insulin resistance and hyperinsulinemia [86]. Insulin resistance and hyperinsulinemia affect fertility in different ways in males and females. 

In males, insulin resistance determines a reduction in sperm glucose uptake and, consequently, a reduction in sperm metabolism and motility [87]. Insulin is an important inhibitor of hepatic Sex Hormone Binding Globulin (SHBG) output [88]. The reduction in levels of SHBG is linked to the reduction of total testosterone, which is mainly associated with fewer spermatozoa [80,89]. Moreover, hyperinsulinemia and hyperglycemia are associated with high levels of oxidative stress and reduced levels of antioxidant defenses; this condition determines an alteration in sperm glucose metabolism [90], which is a fundamental source of energy for spermatozoa [91]. On one hand, sperm motility is decreased in these patients; on the other hand, sperm apoptosis is increased [81]. This scenario is linked to male infertility.

In females, hyperinsulinemia is linked to a hyperandrogenism status [92]. In detail, insulin seems to stimulate theca cells to produce androgens [93]. Insulin can bind the insulin growth factor-1 (IGF-1) receptor activating the intracellular pathway that enhances the androgen production in theca cells [94]. Hyperandrogenism can contribute to ovulation disorders, leading also to anovulatory infertility [43,95]. Furthermore, insulin reinforces the activity of LH on granulosa cells [96]. The premature luteinization and, consequently, the follicular arrest determines oligo-anovulation in these women [97].

#### 3.1.3. Proteins

Proteins are found in milk, eggs, meat, and chicken. It is known that a low-protein diet is an important risk factor for male infertility because it causes a reduction in testis weight and testosterone levels [98]. On the contrary, the effects of a high-protein diet are not so clear; it seems that its effects on fertility depend on the sources [99]. Proteins obtained from red meat and poultry increase the levels of IGF-1 in women [100]. Higher IGF-1 levels correlate with ovulatory disorders and anovulatory infertility [101]. The effects of milk products on fertility depend on the various fat contents [102]. On the contrary, the consumption of plant proteins improves insulin sensitivity, reduces IGF-1 levels, and has a positive effect on ovulation [19].

In the Western diet, the consumption of proteins obtained from red meat is higher than the consumption of plant proteins, causing a higher risk of ovulatory disorders.

## 4. Arabic Middle Eastern diet

The dietary habits in Middle Eastern countries have changed during the last fifty years [103]. Sudden economic growth has led to changes in lifestyle and nutritional status. In particular, the discovery of large oil reserves has boosted the economies of Middle Eastern countries such as Saudi Arabia, Iran, Iraq, Kuwait, and Egypt [104]. In these countries, the standard of living has become higher and has been influenced by the Western way of life. Formerly, the traditional diet was rich in seasonal fruits and vegetables and low in cholesterol and fat; nowadays, the diet is more like a Western diet, which is rich in fats, specially SFA, and high glycemic carbohydrates [105]. This dietary change has led to a rapid rise in the prevalence of gestational diabetes in pregnant women and of type 2 diabetes mellitus [106]. Moreover, the prevalence of obesity in the Arabic region is amongst the highest in the world [107]. As reported before, a diet rich in fat and sugar is linked with ovulatory disorders in females and impaired sperm quality in males. In addition, obesity harms fertility both in males and in females. It is easy to deduce that this dietary pattern is linked to an increased risk of infertility. Analyzing some countries, it was observed that in Saudi Arabia the TFR has declined from 7.20 (in 1950) to 2.31 (in 2022), with a relative change of −68%. Moreover, in the United Arab Emirates the TFR has changed from 6.94 (in 1950) to 1.66 (in 2022), with a relative change of −76% [22].

In this scenario, public health policies should define lifestyle modification programs to promote healthy dietary habits [108]. Many studies reported that among the Arabic population, especially in Arabic women, there are sociocultural barriers to a healthy lifestyle [109,110]. To be effective, these programs should be modified according to cultural and religious practices [111]. 

## 5. Asian Diet

As for the Middle Eastern diet, the Asian diet has also rapidly changed in recent years [112]. Globalization, urbanization, and rapid economic growth made a fundamental contribution to the lifestyle modifications of Asian people [113]. The traditional Asian diet was balanced and healthy. It was a diet rich in fibers, vitamins, and antioxidants and low in fats, meat, and dairy foods [114]. This dietary pattern was recommended because it was protective for different diseases, such as diabetes and cardiovascular diseases [115]. 

Nowadays, the Asian diet is characterized by an increased consumption of wheat, animal proteins, and foods rich in fats and sugars [116,117]. Specifically, the consumption of white rice is typical in Asian countries. In the Asian diet there are 3–4 servings a day of white rice, while in the Mediterranean diet there are 1–2 servings a week of white rice [118]. Compared to other carbohydrates (such as bread, brown rice, and pasta), white rice has a higher glycemic index [119]. The increased postprandial levels of insulin and glucose may be associated with higher risk of insulin resistance and type 2 diabetes mellitus [120]. Moreover, different studies have demonstrated an association between the consumption of white rice and metabolic syndrome [121]. 

Currently, the Asian diet is also characterized by increased intake of TFAs and SFAs, which are obtained from palm oil, coconut oil, corn oil, and sesame oil. On the contrary, the intake of PUFAs, obtained from fish oil, is low in this population [112]. In this scenario, as illustrated before, the imbalanced consumption of oils and fats is associated with altered fertility, both in males and in females.

As examined before, a Westernized diet is associated with a higher risk of infertility. For example, in China the TFR has declined from 5.29 (in 1950) to 1.66 (in 2022), with a relative change of −69%. In Japan, the TFR varied from 3.48 (in 1950) to 1.53 (in 2022); in this case, the relative change was −56% [22].

As for Arabic Middle Eastern Countries, lifestyle interventions should be identified in the Asian population to encourage healthy eating habits. 

The principal differences between dietary patterns are summarized in Table 1.

## 6. Other Dietary Patterns

### 6.1. Vegetarian and Vegan Diet

Vegetarian and vegan diets are characterized by the consumption of plant-based foods. The vegetarian diet does not include fish, meat, or poultry, but it includes products derived from animals, such as eggs and dairy products. Conversely, a vegan diet eliminates all animal products. The consequences of a vegetarian or vegan diet on fertility are still debated [122]. From one point of view, the possible lack of some nutrients, such as iron or essential fatty acids, could increase the risk of infertility. Moreover, the vegetarian diet is characterized by soy foods, which are rich in isoflavones [123,124]. Isoflavones have estrogen-like effects on sperm, leading to feminization in men and, consequently, to male infertility [125]. In 2016, Orzylowska et al. compared sperm characteristics between 26 vegetarians, 5 vegans and 443 non-vegetarian males. Vegetarians had significantly lower sperm concentrations (50.7 ± 7.4 million/mL) when compared with non-vegetarians (69.6 ± 3.2 million/mL). Moreover, total motility was lower in vegetarians (33.2 ± 3.8%) than in non-vegetarians (58.2 ± 1.0%). Interestingly, no differences were found for total sperm motility and sperm concentration when they were compared between vegans and non-vegetarians [123]. In this scenario, it appears that the vegetarian diet has a negative effect on male fertility.

From another point of view, both vegetarian and vegan diets are rich in antioxidants. As reported before, antioxidants (carotenoids, vitamin C, vitamin E, flavonoids, and polyphenols) have a positive effect on fertility by reducing oxidative stress and improving sperm and oocyte quality [126,127]. In addition, vegetarians and vegans generally have a normal BMI; so, the negative effects of obesity on fertility are absent in this population [128]. In 2021 Kljajic et al. compared sperm quality in ten vegans and in ten non-vegetarians male. In this study it was found that sperm quality was higher in vegan group. In particular, non-vegetarians had increased DNA denaturation (14.7 [7–33.5] vs. 8.2 [3–19.5]; *p* = 0.05). Moreover, vegan group had a higher percentage of both rapid progressive motile sperm (17.5 [15–30] vs. 1 [0–7]; *p* < 0.0001) and higher sperm concentration (224.7 [117–369] vs. 119.7 [64.8–442.8]; *p* = 0.011) [126]. In contrast to the study conducted by Orzylowska et al., in this study the population cohort was smaller, but the groups were divided equally.

Further studies, with larger and homogeneous sample size, are needed to evaluate the possible link between vegetarian or vegan diets and infertility both in males and in females.

### 6.2. Ketogenic Diet

The ketogenic diet eliminates high-carbohydrate foods and increases high-fat foods. The objective of this dietary pattern is to use ketones from the breakdown of fats as a primary energy source [129,130]. The ketogenic diet has a positive impact on different diseases, particularly epilepsy and neurodegenerative diseases [131].

The effects of a ketogenic diet on fertility are still debated. On one hand, in certain populations, such as obese people, the weight loss linked to this diet positively influences fertility [132]. Mavropoulos et al. analyzed a cohort of women with BMI > 27 kg/m^2^ with PCOS and demonstrated that a ketogenic diet reduced total body weight, reduced risk of hyperinsulinemia, and reduced both LH/FSH ratio and free testosterone levels [132]. As reported before, a diet low in carbohydrates reduces insulin production and, consequently, hyperinsulinemia and insulin resistance, which are observed in most infertile women and men [133]. Furthermore, compared to other hypocaloric diets, a ketogenic diet is linked with increased levels of SHBG, reduced testosterone levels and reduced LH/FSH ratio in women [134]. Recently, a study analyzed twelve PCOS-positive women with a previous failed IVF cycle. These patients followed a ketogenic diet for 14 ± 11 weeks. When analyzing this cohort before and after the nutritional intervention, it was found that there was significant weight loss (−7.9 ± 1.1 kg) and significant improvements in implantation (83.3% compared to 8.3% before ketogenic diet), in clinical pregnancy (66.7% compared to 0% before the ketogenic diet), and in live birth rates (66.7% compared to 0% before the ketogenic diet). There was no difference found for oocyte number, fertilization rate, or viable embryos produced [135].

On the other hand, the ketogenic diet does not consider its fat sources. This dietary pattern often induces high consumption of SFA and cholesterol, which increases the risk of infertility both in males and in females with mechanisms that we have already examined [136]. In 2010, a study conducted on mice demonstrated that a diet rich in fats is linked with a higher risk of anovulation and a decreased fertilization rate compared to a standard diet. Mice ovaries were analyzed, and findings suggested that the presence of lipid deposits may be associated with ovarian toxicity and consequent infertility [137]. Moreover, this diet is effective for short-term weight loss, but the long-term effects are still unclear [138].

Further research is required to better understand the role of the ketogenic diet in possible fertility treatment.

## 7. Conclusions

The relationship between fertility and diet is largely analyzed in the literature. Different studies suggested the importance of diet and the contribution of different nutrients in reducing the risk of infertility both in males and in females. We analyzed and discussed how a healthy diet can improve fertility and, on the contrary, how an unhealthy diet can increase the risk of infertility. In contrast to other studies, we also evaluated the dietary habits of the Asian and the Arabic Middle Eastern populations. Additionally, we focused our attention on the vegetarian, vegan, and ketogenic diets. This is the first study presenting a broad vision of dietary patterns and fertility. Despite this, we are still far from a complete picture of the role of single nutrients on fertility.

A balanced diet could have positive effects on the prevention and treatment of different diseases, such as type 2 diabetes mellitus and cardiovascular disease. Currently, clear guidelines on supplementation or diet to enhance fertility are missing.

Further research should solidify the association between diet and fertility and should clarify the impacts of certain dietary patterns on fertility. Only in this way will we be able to make new recommendations on healthy dietary habits, especially in fertile people.

## 8. Take Home Message

Lifestyle has a key role in fertility. Different dietary patterns can positively or negatively influence fertility both in males and in females. As analyzed before, a diet rich in saturated fatty acids, cholesterol, animal proteins, and carbohydrates with a high glycemic index is highly associated with male and female infertility. On the contrary, a diet rich in plant proteins, vegetables, fruits, and antioxidants (carotenoids, vitamin C, vitamin E, flavonoids, and polyphenols) has a positive effect on fertility. In this scenario, it appears that the crucial role of public health policies, especially in developing countries, is to promote healthy dietary patterns and to improve the total fertility rate worldwide.

## Figures and Tables

**Table 1:** Dietary patterns and fertility.

Mediterranean Diet	Western Diet	Arab Middle Eastern Diet	Asian Diet
PUFA: (1) role in endometrial function (2) anti-inflammatory function (3) increased sperm motility, concentration, and volume (4) EVOO is linked with better embryo development	SFA: (1) altered sperm motility and capacitation (2) reduced energy metabolism of spermatozoa (3) ovulation disorders	SFA: (1) altered sperm motility and capacitation (2) reduced energy metabolism of spermatozoa (3) ovulation disorders	SFA e TFA: (1) altered sperm motility and capacitation (2) reduced energy metabolism of spermatozoa (3) ovulation disorders
Carbohydrates: (1) whole grains have a controversial link with a live birth rate (2) reduced glycemic index is correlated with reduced hyperinsulinemia and reduced insulin resistance	Carbohydrates: (1) a high glycemic index is associated with insulin resistance and hyperinsulinemia (2) hyperinsulinemia leads to a reduction of sperm motility and metabolism (3) hyperinsulinemia leads to hyperandrogenism and ovulatory disorders	Carbohydrates: (1) high glycemic index (2) hyperinsulinemia leads to a reduction of sperm motility and metabolism (3) hyperinsulinemia leads to hyperandrogenism and ovulatory disorders	Carbohydrates: (1) white rice has high glycemic index (2) higher risk of hyperinsulinemia and hyperglycemia (3) increased risk of T2DM
Protein: (1) vegetarian protein reduces the risk of anovulatory infertility (2) protein in fish and white meat increases the chance of blastocyst formation (3) red meat (and AGE) can determine ovarian aging, ovarian dysfunction, altered sperm motility, and capacitation	Protein: (1) their effect depends on the source (2) animal protein correlates with higher IGF-1 and a higher risk of anovulatory (3) plant protein reduces IGF-1 levels and improves fertility		
Micronutrients: (1) vitamins E and C have antioxidant effects (2) carotenoids, flavonoids, and polyphenols have antioxidant and anti-inflammatory functions	Cholesterol: (1) altered sperm motility and capacitation (2) decreased testosterone concentration (and reduced sperm concentration)		
Fruits & vegetables: (1) controversial link with live birth, pregnancy, and implantation			

## Data Availability

Not applicable.
